# 1440. Local Antibiogram for *Mycobacterium abscessus* Shows Variability from Previously Published Susceptibility Data

**DOI:** 10.1093/ofid/ofac492.1269

**Published:** 2022-12-15

**Authors:** Timothy McElroy, Richard Starlin, Trevor C Van Schooneveld

**Affiliations:** University of Nebraska Medical Center, Omaha, Nebraska; University of Nebraska Medical Center, Omaha, Nebraska; University of Nebraska Medical Center, Omaha, Nebraska

## Abstract

**Background:**

*Mycobacterium abscessus* complex (MabsC) causes a wide variety of human disease and is frequently resistant to multiple agents. Empiric therapy is typically based on previously published susceptibility data which suggests amikacin (AMK), cefoxitin (FOX), clarithromycin (CLR), and imipenem (IPM) should be utilized. However, there is considerable variability in susceptibility patterns according to locale. We generated an antibiogram based on local MabsC isolates and compared it to previously reported susceptibility data to determine if local variation exists and assist in empiric therapy choice.

**Methods:**

Non-duplicate local isolates of MabsC from 2011-2021 with in-vitro susceptibility data (via broth microdilution per CLSI guidance) available were included in the generation of a local antibiogram. This included standard 14 day incubation for inducible macrolide resistance. A review of the literature via PubMed search was undertaken to generate comparator susceptibility patterns with a focus on published reports with greater than 20 isolates.

**Results:**

Sixty one unique local isolates of MabsC were identified, 44 of which were from lung, eight skin/soft tissue, five bone/joint, two blood, one urine, and one ascitic fluid. Susceptibilities are shown in Table 1. Twenty one manuscripts described susceptibility patterns with a range of isolates from 20 to 404 with a median of 70. These revealed susceptibility ranges for AMK range from 45-100%, FOX 0-72%, CLR 19-96%, and IPM 0%-71% as seen in Table 2.

Susceptibilities of Mycobacterium abscessus isolates

**Figure:**
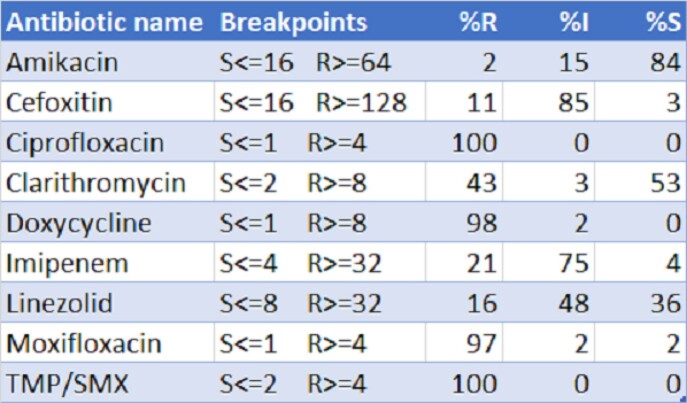

Results of antimicrobial susceptibility testing for 61 isolates of MabsC with percent resistant, intermediate, and susceptible shown, along with break points used per CLSI

Comparison of Drug Susceptibility Rates from Previous Studies

**Figure:**
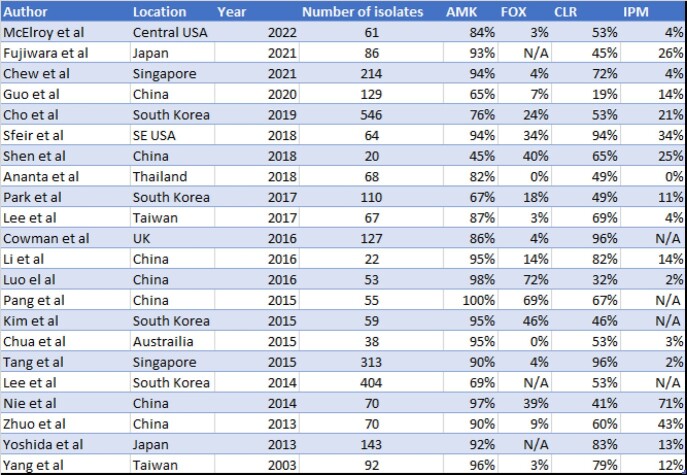

**Conclusion:**

We found high local resistance rates to cefoxitin and imipenem in contrast to empiric therapy recommendations and greatest susceptibility to amikacin. Published data shows a wide variation of susceptibility patterns to the recommended therapies based on location. It would be clinically advantageous for institutions to generate an antibiogram based on their local resistance data to assist empiric therapy choices in MabsC infections. Our data suggests that such local antibiograms could lead to substantial changes from the recommended empiric therapy at the institutional level for these infections which are historically challenging to treat.

**Disclosures:**

**Trevor C. Van Schooneveld, MD**, bioMerieux: Advisor/Consultant|bioMerieux: Grant/Research Support|Insmed: Grant/Research Support|Merck: Grant/Research Support|Thermo-Fischer: Advisor/Consultant.

